# Water Absorption and Distribution in a Pultruded Unidirectional Carbon/Glass Hybrid Rod under Hydraulic Pressure and Elevated Temperatures

**DOI:** 10.3390/polym10060627

**Published:** 2018-06-07

**Authors:** Chenggao Li, Guijun Xian, Hui Li

**Affiliations:** 1 Key Lab of Structures Dynamic Behavior and Control, Ministry of Education, Harbin Institute of Technology, Harbin 150090, China; lichenggao02@126.com (C.L.); lihui@hit.edu.cn (H.L.); 2 Key Lab of Smart Prevention and Mitigation of Civil Engineering Disasters of the Ministry of Industry and Information Technology, Harbin Institute of Technology, Harbin 150090, China; 3 School of Civil Engineering, Harbin Institute of Technology, Harbin 150090, China

**Keywords:** hybrid, FRP rod, water absorption, hydraulic pressure, Fickian law

## Abstract

A pultruded unidirectional carbon/glass reinforced epoxy hybrid FRP rod with 19 mm of diameter was developed for a sucker rod and lifting oil wells. The rod possesses a 12-mm diameter carbon fiber core and a 3.5-mm thick outer shell. The rod was exposed to high-temperature immersion in water under hydraulic pressure. To understand the long-term service performance of the rod, immersions in water at 20 °C, 40 °C, or 60 °C under 20 MPa of pressure for 1 year were conducted on the water uptake and distribution in the rod. The water uptake data were fitted by Fickian diffusion law, and the diffusion coefficient and the maximum water uptake were derived. Water distribution in the rod as a function of the immersion time, temperature, and hydraulic pressure was analyzed theoretically. This study revealed the accelerating effects of the elevated temperatures and the hydraulic pressure on the water diffusion in the hybrid rod.

## 1. Introduction

Fiber-reinforced polymer (FRP) composites possess many advantages, e.g., low-weight, high specific strength and specific stiffness, convenience of installation, and superior fatigue resistance, especially for carbon FRPs (CFRPs) [1]. In recent year, FRPs have been widely used in various industry fields as a competitive alternative to traditional materials, such as steel [2]. 

Generally, FRP rebar or rods are commonly used as the internal reinforcement to replace the steel bar for concrete beam and bridge decks [3]. However, the relatively lower tensile modulus and ultimate elongation, as seen in glass fiber-reinforced polymer (GFRP) and basalt fiber-reinforced polymer (BFRP), greatly limited their application. To make full use of the tensile properties and long-term durability of FRPs, recently, FRPs have found some new applications, such as sucker rods for lifting oil wells, submarine structures, subsea oil industry use, and as oceanographic profilers as load-bearing bars or rods to replace the steel materials [4,5,6]. For such novel applications, the long-term properties of FRPs under complex service conditions (e.g., high temperatures, hydraulic pressure, etc.) are the main concerns and need to be fully understood [7]. Furthermore, the degradation mechanisms and service lives of FRPs under the coupling effect of multiple environments are necessary to know for the development of advanced FRPs and safe design [8]. 

In the past two decades, water/moisture uptake and diffusion in FRPs and their influence on the degradation of mechanical properties were well investigated [9,10,11,12]. However, the moisture/water uptake coupled with hydrostatic pressure cannot be established completely so far. Some contradictory results were reported, e.g., an increasing effect [13], no effect [14], or a reducing effect [15,16] on the water uptake. Pollard et al. [17] established a linear relationship between the hydraulic pressure and moisture content in a glass-fiber-reinforced polyester FRP. Whitaker et al. [18] found that the hydraulic pressure reduced the diffusion coefficient only when the temperatures were over 25 °C and the moisture saturation level was not influenced. Davies et al. [13] tested a filament-wound, carbon-fiber-reinforced epoxy under a hydrostatic pressure of 100 bar at 60 °C for 3.5 years and found a significant rise in the saturation level with the pressure. It is worth noting that these studies did not set up quantitatively the influence of the hydrostatic pressure and the water diffusion. 

Among FRPs, glass-fiber-reinforced polymer (GFRP) composites are most widely used for various structures due to low cost. However, GFRPs have relatively low tensile strength and modulus and are prone to degrade owing to hydrolysis, plasticization, and swelling of matrices and debonding of fiber/matrices when exposed to hydrothermal conditions [19,20]. By contrast, carbon fiber reinforced polymer (CFRP) composites possess excellent chemical and corrosion resistance, and higher modulus and strength, but lower strain at break [21,22,23,24,25]. However, the high cost of CFRPs is a serious drawback and limits its applicability in many engineering structures. To balance their advantages, the hybrid FRPs with glass and carbon fibers are well accepted [26]. 

So far, limited investigations were reported on the long-term durability of hybrid FRP composites [27]. Barjasteh et al. [8] studied the moisture absorption of hybrid rods with a diameter of 7.75 mm. In the work, 1D and 2D moisture diffusion models were calculated to obtain the radial and longitudinal moisture diffusivity coefficients. In addition, a complex intermingling of GF/CF interface acting as a temporary moisture barrier was reported. Tsai et al. [27] experimentally studied moisture uptake behavior of a hybrid FRP rod (for conductor core) after immersion in water at different temperatures up to 32 weeks. Fickian law describes well only the initial water uptake. No cracks were found when the water uptake was less than saturation. In summary, the mechanical and physical properties of hybrid FRP composites were reported to be adversely affected by the long-term water absorption process when exposed to high temperature and moisture [28,29,30].

The aim of this work is to study the coupling effect of immersion temperature and hydrostatic pressure on the water diffusion and distribution in the hybrid FRP rod. The water uptake test was performed at 20 °C, 40 °C, and 60 °C with or without hydraulic pressure. The Fickian law was applied to describe the water uptake behavior and obtain the average diffusivity coefficient. Furthermore, the water distribution in the rod was obtained through theoretical analysis. The effects of the immersion temperature and hydrostatic pressure were investigated quantitatively. 

## 2. Materials and Methods

### 2.1. Raw Materials

**Figure 1:**
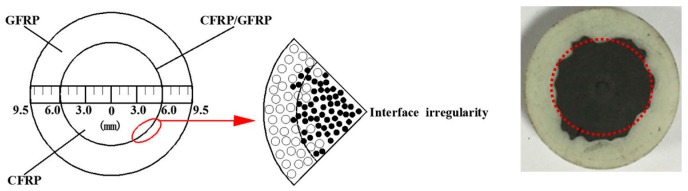
Cross-section of the hybrid HFRP rod.

The round hybrid FRP rod with a diameter of about 19 mm is shown in Figure 1. It includes two layers, a glass fiber shell layer with a thickness of approximately 3.5 mm and carbon fiber core with a radius of approximately 6 mm (nearly a circle). The rod was manufactured via pultrusion technique with a kind of bisphenol-A epoxy resin matrix (Airstone^TM^ 1122E, Blue Cube Chemicals Company, Zhangjiagang, China).

### 2.2. Water Uptake Test

**Figure 2:**
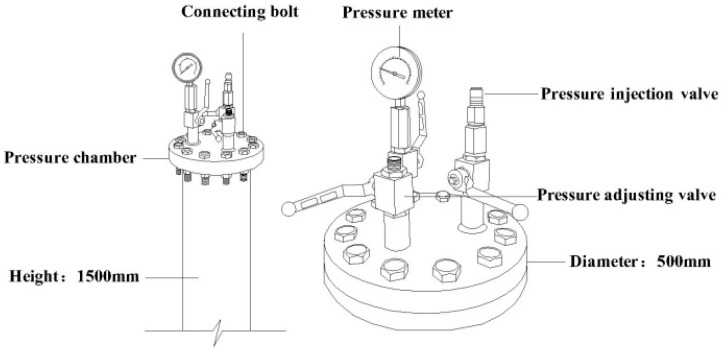
Immersion cylinder with hydraulic pressure.

Figure 2 shows the steel cylinder for hydraulic pressure immersion, which was made by a steel tube with a thickness of 10 mm attached with pressure meter (showing the water pressure) and hydraulic valves (adjusting and controlling the pressure value). The heating and insulation setups including the glass fiber heating band, insulation wool, voltage regulator, and thermocouple, were wound around the surface of the steel tube. With this cylinder, the hydraulic water pressure of 20 MPa was realized with a high-pressure injection syringe system (3600, Tenglong Cleaning Machine Company, Taizhou, China). The cylinder was heated by the wrapped heating tapes, and the temperature was controlled by ±1 °C. The aging condition of 60 °C and 20 MPa hydraulic pressure was designated as “60 °C/20 MPa”. It was noted that the selected aging temperature (60 °C) was generally accepted and studied by the peers as shown [13,27], and the hydraulic pressure was determined as 20 MPa to reflect the more practical service condition [7,13]. 

**Figure 3:**
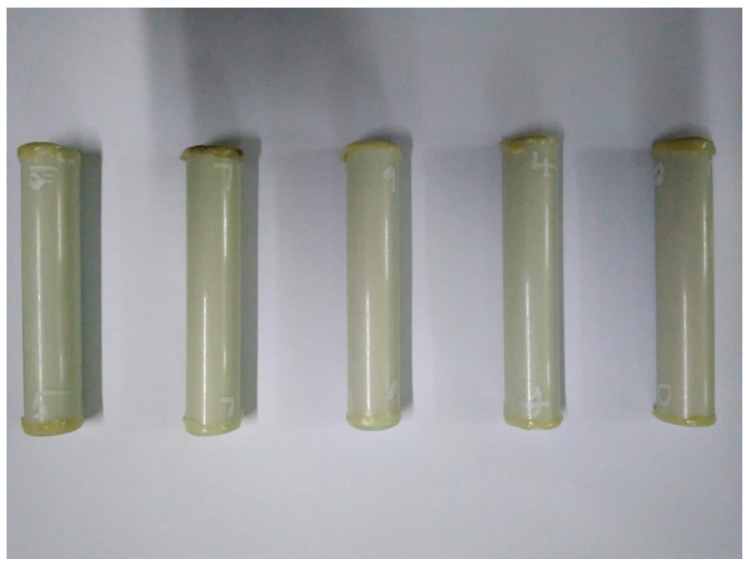
Rod specimens with the length of 100 mm for water absorption. Note, the ends were capped with an epoxy adhesive.

The FRP rod was cut into about 100 mm in length for the water absorption test according to ASTM D5229. Ten samples were prepared for each condition. To model the radius diffusion, the ends of rod samples were capped using an epoxy adhesive shown in Figure 3. Then the capped samples were put in an oven at 60 °C for 2 days. On the one hand, this action was to make the epoxy adhesive cure sufficiently when considering its glass transition temperature was 89.9 °C. On the other hand, the retained surface moisture and low-molecular-weight species can be removed effectively. It was noted that the drying time (2 days) was enough, because the longer drying led to the matrix degradation and removal of sizing just as in the observation by Barjasteh [8]. Lastly, the rods were placed in the thermostatic water baths (20 °C, 40 °C, and 60 °C) or immersion cylinder with hydraulic pressure (60 °C/20 MPa) for water absorption. Distilled water at 20 °C, 40 °C, and 60 °C was used as the immersion media. Samples were removed from immersion and dried with tissue periodically to measure the mass. Samples were re-immersed after mass measurement as soon as possible. An electronic balance with an accuracy of 0.1 mg was used for the mass measurement. 

## 3. Results and Discussion

### 3.1. Water Absorption and Diffusion in the Hybrid Rod

**Figure 4:**
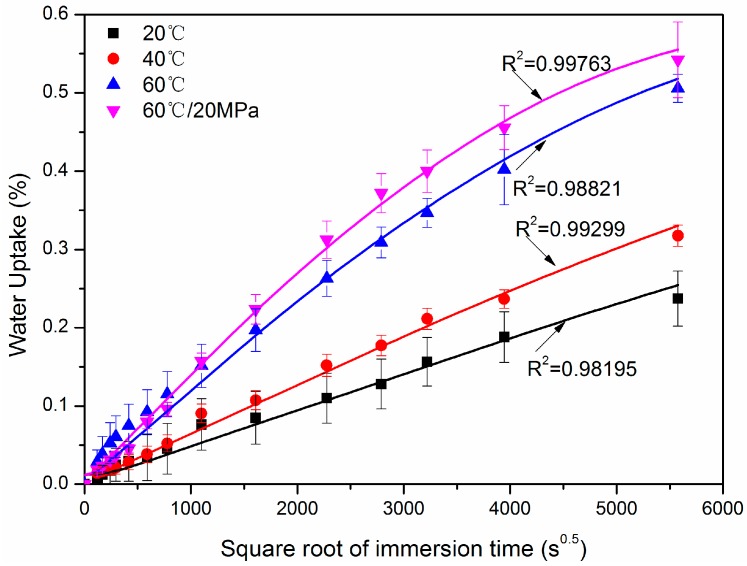
Water uptake versus the square root of time for hybrid rods exposed to 20 °C, 40 °C, 60 °C, and 60 °C/20 MPa.

Figure 4 shows the water uptake versus the square root of immersion time at 20 °C, 40 °C, 60 °C, and 60 °C with hydraulic pressure of 20 MPa for one year. Each discontinuous point represented the average weight gain for ten samples and was obtained by the following equation:

(1) $$W(\%)=\frac{W_{t}-W_{0}}{W_{0}}\times 100\%$$

where *W*(%) was the weight gain, *W_t_* was the wet weight at time *t*, and *W*_0_ was the dry weight at *t* = 0. After one year of immersion, the maximum percent of weight gain for 20 °C, 40 °C, 60 °C, and 60 °C/20 MPa were 0.24% (±0.035%), 0.32% (±0.014%), 0.51% (±0.018%), and 0.54% (±0.048%), respectively. It was noted that the values in the bracket showed the standard deviations of each condition. From the slope of the water absorption curve, the decreasing water absorption rate was observed for the case of 60 °C and 60 °C/20 MPa, while a linear dependence of the weight gain versus the square root of immersion time was determined for 20 °C and 40 °C immersions. 

It is interesting to see that the hydraulic pressure increased the water uptake after a certain time of immersion (Figure 4). A recent study indicated that hydraulic pressure brought in more moisture uptake for glass-fiber-reinforced epoxy hand layup plates [7]. The study suggested that the hydraulic pressure helps more water molecules diffuse into the void of the FRPs, formed during FRP manufacturing. In the current study, the rod was produced through a pultrusion process, and low void content was expected. Therefore, the water uptake was increased to a small extent with the pressure. 

To obtain the water absorption parameters of the hybrid FRP rod, the Fickian diffusion law was adopted [26]:

(2) $$\frac{M_{t}}{M_{\infty }}=1-\sum_{n=1}^{\infty }\frac{4}{R^{2}\alpha_{n}^{2}}\exp(-D_{av}\alpha_{n}^{2}t)$$

where *M_t_* was the weight gain in time *t*, *M*_∞_ was the saturation level of water absorption, *R* was the radius of the hybrid rod, *α*_n_ was the *n*th root of the zero-order Bessel function, and *D_av_* was the average diffusivity coefficient of the hybrid rod.

For the FRP composites, the voids inside the material were the main water-storage pathway. When there was no cracking mechanism acting on the FRP from sustained load and elevated temperature, the water storage capacity (saturation water uptake *M*_∞_) inside FRP material remained unchanged, irrelevant to the immersion temperature and time [27,31]. In this paper, there was no sustained load and the exposed temperature (60 °C) was not enough to lead to any cracking. Constant saturation water uptake may be appropriate for the current four immersion cases. It was noted that the data of the weight gain at 60 °C is only available for maximum immersion times (5577 s^0.5^) from the water absorption tests. For the unavailable weight gain at greater immersion times, a speculative constant value (0.6) is assumed for the saturation water uptake based on the maximum weight gain at 60 °C and decreasing water absorption rate (Figure 4). With this value, fittings were performed using the *OriginPro* 8.1 software (OriginLab Corporation, Northampton, MA, USA), and the diffusion parameters were determined, shown in Table 1. 

As shown in Figure 4, the R-squared of four curve fittings were not less than 0.98, indicating that the water uptake of the hybrid rod was well conformed to the Fickian law. Table 1 shows that *D_av_* increased gradually with the immersion temperature as expected and were frequently reported [31]. As regards to the hydraulic pressure, the diffusion coefficient in this study increased about 37% compared to pressure-free immersion. 

**Figure 5:**
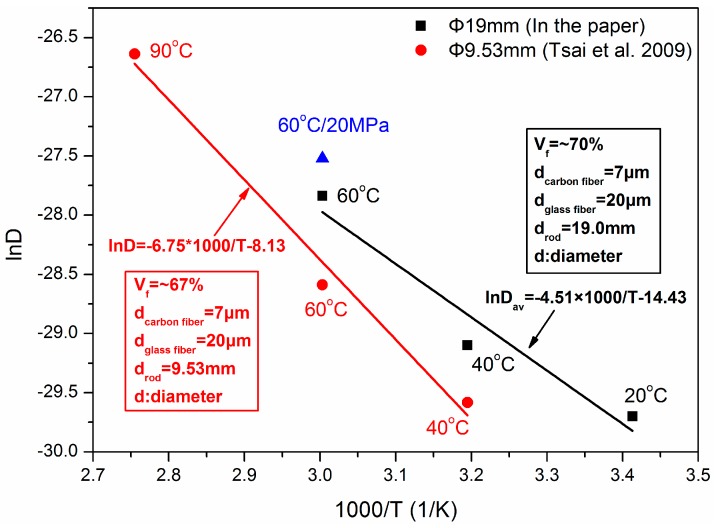
Average diffusivity coefficient vs. temperature of GFRP–CFRP hybrid rods.

To analyze the dependence of the average diffusivity coefficient on the immersion temperature, Figure 5 presents the relationship of ln *D* ~ 1000/*T*. In addition, literature data on a low-diameter GFRP–CFRP rod was plotted for comparison [27]. The diameters of the CFRP core and hybrid rod were 7 mm and 9.53 mm, respectively. The global fiber volume content was 67 vol %. As found, the diffusion coefficients in this study were higher than Tsai et al.’s results at the same immersion temperatures, i.e., 40 °C and 60 °C. The deviation is attributed to some initial defects inside the current high-diameter rod, such as resin matrix void, fiber microcrack, or fiber/resin interface imperfection. The initial defects probability increased with the diameter of the rods, bringing in more water uptake. The higher diffusion coefficient in this study, therefore, is attributed to a greater possibility of defects formed due to the larger diameter.

### 3.2. Water Concentration Distribution in the Hybrid Rod

After obtaining the diffusion coefficient, the water concentration distribution as a function of radial position in the hybrid rod was deduced through the diffusion mathematics theory and the different diffusivity coefficients of shell/core layer were considered to revise the average diffusivity coefficient *D_av_* in Equation (2). 

In this study, the hybrid rods were capped at both ends using an epoxy adhesive to model the radius diffusion of the water molecules. For a long cylinder in which diffusion was everywhere radial, the diffusion equation was shown as the following [32]:

(3) $$\frac{\partial C(r,t)}{\partial t}=\frac{1}{r}\frac{\partial }{\partial t}(rD\frac{\partial C(r,t)}{\partial r})$$

where *C*(*r,t*) was the concentration distribution, *r* was the radial position of the hybrid rod, *t* was the water absorption time, and *D* was the diffusivity coefficient; its value was *D_C_* for the CFRP core layer and *D_G_* for the GFRP shell layer. 

For the hybrid rod, the boundary condition was shown as the following:

(4) $$\begin{array}{l} C=C_{0},r=b,t\geq 0\\ C=C_{1},a<r<b,t=0 \end{array}$$

The detailed derivations are presented in the Appendix. Finally, the concentration distribution of water uptake for the hybrid rod is obtained and expressed as the following:

(5) $$C_{C}(r,t)=1-\frac{2}{a}\sum_{n=1}^{\infty }\frac{J_{0}(\alpha_{n}r)}{J_{1}^{}(a\alpha_{n})\alpha_{n}}\frac{1}{\alpha_{n}}\exp(-D_{C}\alpha_{n}^{2}t)),\;0<r<a$$

(6) $$C_{G}(r,t)=1-2\sum_{n=1}^{\infty }\frac{J_{0}(\alpha_{n}r)(bJ_{1}(b\alpha_{n})-aJ_{1}(a\alpha_{n}))}{b^{2}J_{1}^{2}(b\alpha_{n})-a^{2}J_{1}^{2}(a\alpha_{n})}\frac{1}{\alpha_{n}}\exp(-D_{G}\alpha_{n}^{2}t)),\;a<r<b$$

where *C_C_*(*r,t*) and *C_G_*(*r,t*) were the concentration distributions of the CFRP core and GFRPs shell layer, respectively; *a*, *b* were the radii of the core layer CFRP and the whole rod, respectively; *J*_0_, *J*_1_ were the zero and first-order Bessel function of the first kind, respectively; and *D_C_*, *D_G_* were the diffusivity coefficients of CFRP and GFRP, respectively. 

For the Equations (5) and (6), the interface concentration *C_I_* of CFRP/GFRP should satisfy the continuous condition (namely, *C_I_* = *C_C_* = *C_G_*, *r* = *a*) owing to the progress of water diffusion from shell layer to core. Besides, the boundary concentration at *r* = *b* was normalized to be one. 

According to Equations (5) and (6), to determine the concentration distribution in the shell layer and core in the hybrid rod, the diffusivity coefficients *D*_C_, *D*_G_ need to be known. Barjasteh et al. [8] experimentally obtained the diffusivity coefficients *D_C_*, *D_G_* of CFRP and GFRP in a hybrid rod with a diameter of 7.75 mm and the fiber volume fraction of ~67%. As reported, the diffusivity coefficient largely relied on the interface area of fiber/resin. Increasing the interface areas improved the probability of imperfect fiber-resin bonding and microvoids, providing more paths for water molecule diffusion [33]. Specifically, the diffusivity coefficient was mostly dependent upon the diameter and volume content of fibers, when ignoring the variety of fiber and resin. The diffusivity coefficient (*D_av_* shown in Equation (2)) of the hybrid rod can be viewed as a linear combination of the diffusivity coefficient *D_C_* and *D_G_* [33]. The present hybrid rod has the same diameter of carbon fibers (7 μm) and glass fibers (20 μm) and similar volume content of fiber, ~67% to E. Barjasteh et al.’s samples and ~70% in this study. After the acquisition of the average diffusivity coefficient of the hybrid rod, the diffusivity coefficient of each component was determined as follows:

(7) $$D_{av}=AD_{C}+(1-A)D_{G}$$

(8) $$B=\frac{D_{C}}{D_{G}}$$

where *A* was the linear combination coefficient for the hybrid and non-hybrid rod, and *B* was the scale factor relevant to diameter and volume content of the fiber. From the result reported by Barjasteh et al. [8], *A* and *B* were determined to be 0.6 and 1.32, respectively. Through Equations (2), (7) and (8), the diffusivity coefficients *D*_C_ of CFRP and *D*_G_ of GFRP exposed to 20 °C, 40 °C, 60 °C, and 60 °C/20 MPa were obtained as shown in Table 2. 

As shown in Equations (5) and (6) and Table 2, the water concentration distribution of the hybrid rod was mainly dependent on the radial position *r*, immersion time *t*, and exposed temperature *T* (diffusivity coefficient *D*), and their influences are discussed below.

**Figure 6:**
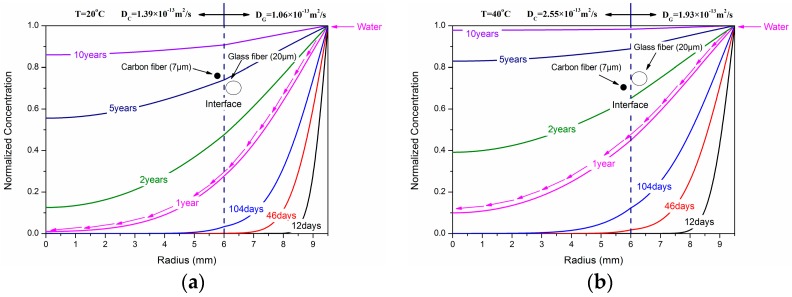

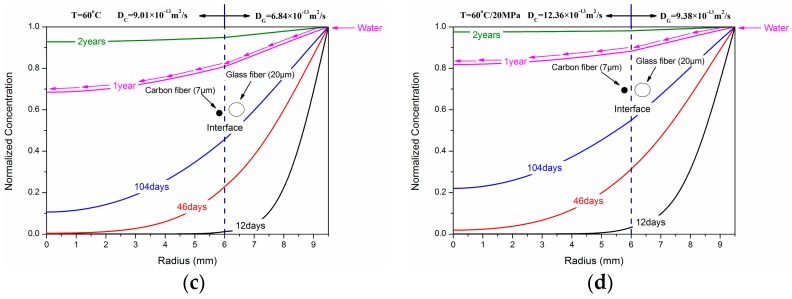
Dependence of water concentration distribution on the radial position of (**a**) 20 °C, (**b**) 40 °C, (**c**) 60 °C, and (**d**) 60 °C/20 MPa.

**Figure 7:**
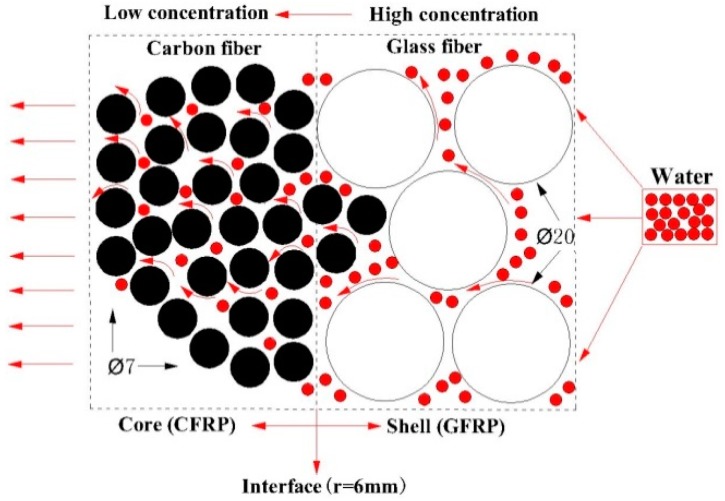
Schematic diagram of water absorption behavior of hybrid rod.

Figure 6 shows the dependence of water concentration distribution on the radial position exposed at 20 °C, 40 °C, 60 °C and 60 °C/20 MPa. It can be seen that when the immersion time was one year, the normalized concentration of CFRP/GFRP interface exposed at 20 °C and 40 °C was 0.28 and 0.45, respectively, while its value was 0.81 and 0.88 for 60 °C and 60 °C/20 MPa, respectively. Figure 7 shows the schematic diagram of water absorption behavior of the hybrid rod. As shown, the water molecules diffused from shell layer (GFRP) to core layer (CFRP) owing to the concentration difference of two-phase media until the saturated state reached when the normalized concentration at *r* = 0 mm was one.

**Figure 8:**
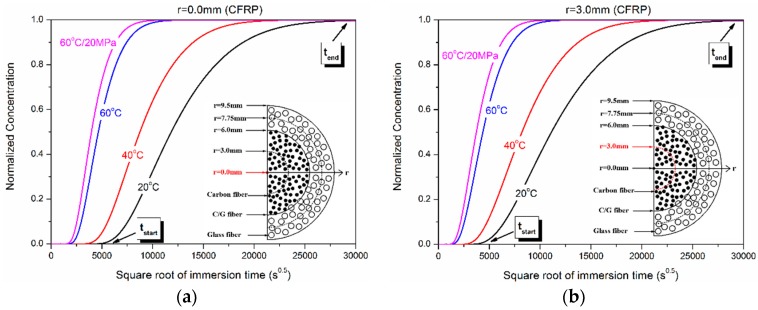

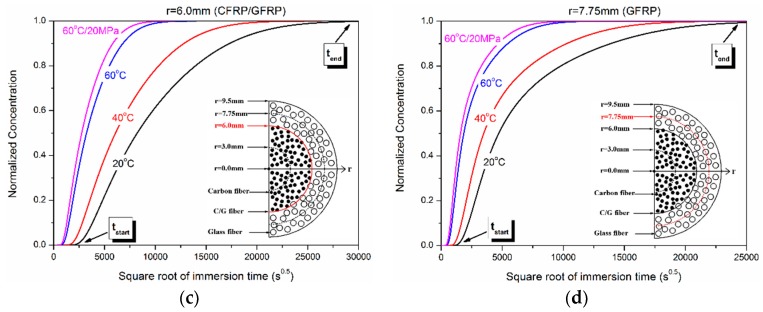
Dependence of water concentration distribution on the immersion time of (**a**) *r* = 0.0 mm, (**b**) *r* = 3.0 mm, (**c**) *r* = 6.0 mm, and (**d**) *r* = 7.75 mm.

Figure 8 shows the dependence of water concentration distribution on the immersion time at different radial positions (*r* = 0, 3.0 mm, 6.0 mm, and 7.75 mm). As shown, the concentration difference at different immersion temperatures gradually decreased with the increase of radial position. This illustrated that the water absorption acceleration action relevant to exposed temperature had a retardation effect along the radial position. In addition, hydraulic pressure had a certain contribution to the water absorption concentration. 

Table 3 shows the immersion time (*t*_start_, *t*_end_) of initial and saturated normalized concentration exposed at 20 °C, 40 °C, 60 °C, and 60 °C/20 MPa. For convenience, *t*_start_ was the immersion time when the normalized concentration was more than zero, letting *C* = 0.01 for convenience, and *t*_end_ was the immersion time when the normalized concentration was to reach one, letting *C* = 0.99. It should be pointed out that *t*_end_ was also the saturated time for *r* = 0 mm owing to the disappeared concentration difference, denoted by A*. As shown, *t*_start_ and *t*_end_ were decreased with the increase of immersion temperature and radial position. The water absorption saturated time of the hybrid rod was 21.4 years, 11.8 years, 3.3 years, and 2.4 years at 20 °C, 40 °C, 60 °C, and 60 °C/20 MPa, respectively. 

**Figure 9:**
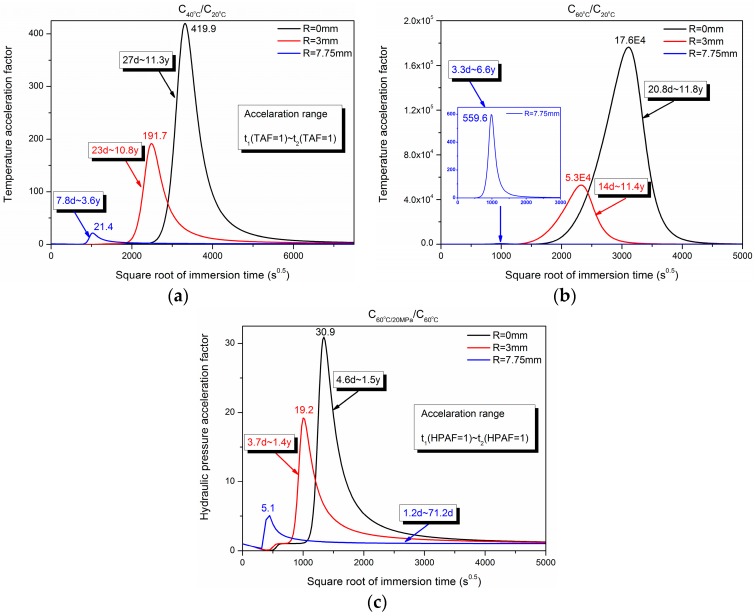
Dependence of temperature (TAF) and hydraulic pressure acceleration factor (HPAF) and range on the immersion time of (**a**) C_40°C_/C_20°C_, (**b**) C_60°C_/C_20°C_, (**c**) C_60°C/20MPa_/C_60°C_.

The temperature acceleration factor (TAF) and hydraulic pressure acceleration factor (HPAF) on the water uptake were obtained through taking the concentration distribution of exposures at 20 °C and 60 °C as the reference value and is shown in Figure 9. For *r* = 0.0 mm, the maximum temperature accelerate factors were 419.9 and 17.6 × 10^4^ for C_40°C_/C_20°C_ and C_60°C_/C_20°C_, respectively. Similarly, the maximum hydraulic pressure acceleration factor was 30.9 at *r* = 0.0 mm.

Table 4 shows the maximum temperature and hydraulic pressure acceleration time for *r* = 0, 3.0 mm, and 7.75 mm. The higher immersion temperature and larger radial position led to the shorter acceleration time, such as *t* = 11.0 d for C_60°C_/C_20°C_ at *r* = 7.75 mm. Compared to the maximum temperature acceleration time, the hydraulic pressure acceleration time was relatively smaller (2.3 d~20.8 d). 

**Figure 10:**
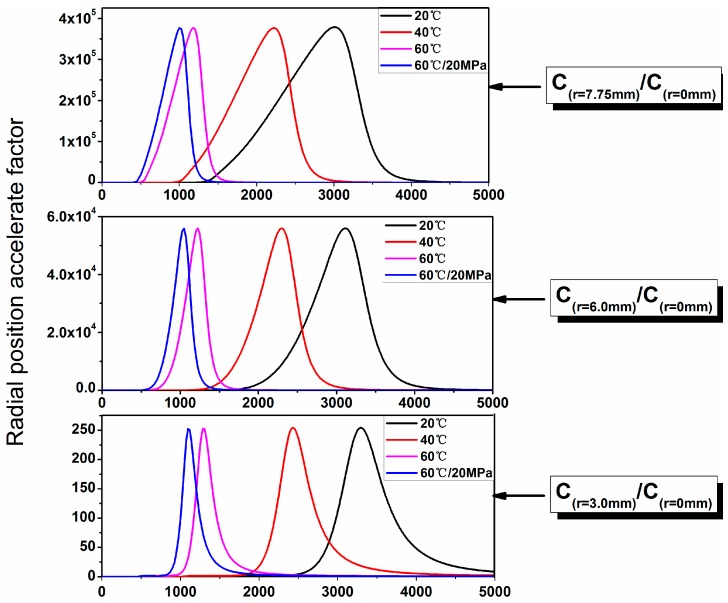
Dependence of radial position acceleration factor (RPAF) on the immersion time.

The radial position acceleration factor (RPAF) was obtained in Figure 10. For the same radial position, the temperature and hydraulic pressure had an almost identical acceleration factor. The maximum RPAF decreased with the movement to the core layer of radial position. In addition, the maximum radial position acceleration time increased with the decrease of immersion temperature. 

**Figure 11:**
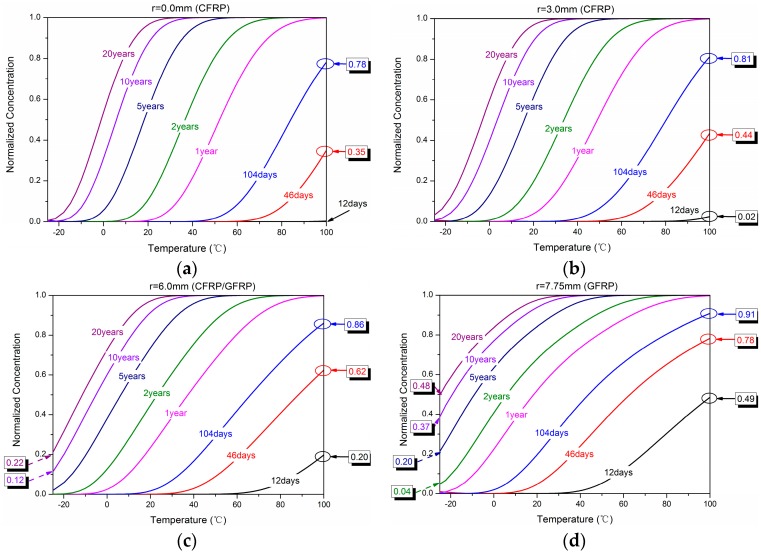
Dependence of water concentration distribution on the immersion temperature of (**a**) *r* = 0.0 mm, (**b**) *r* = 3.0 mm, (**c**) *r* = 6.0 mm, and (**d**) *r* = 7.75 mm.

**Figure 12:**
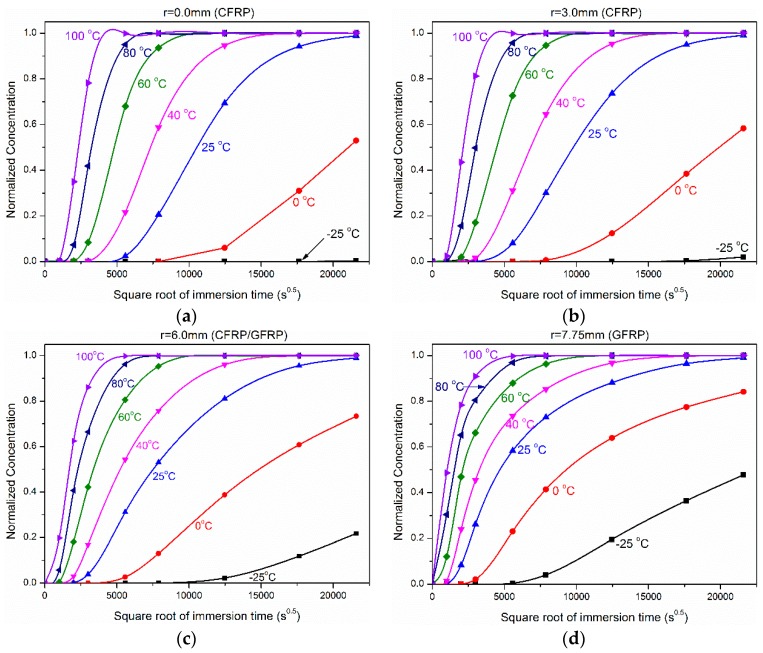
Dependence of water concentration distribution on the immersion time from −25 °C to 100 °C for (**a**) *r* = 0.0 mm, (**b**) *r* = 3.0 mm, (**c**) *r* = 6.0 mm, and (**d**) *r* = 7.75 mm.

Figure 11 shows the influence of immersion temperature on the water absorption concentration. Figure 12 shows the dependence of water concentration distribution on the immersion time from −25 °C to 100 °C. It should be noted that the minimum and maximum prediction temperatures were −25 °C and 100 °C, respectively, to simulate the freezing and evaporation conditions. It was obvious that the concentration increased quickly with immersion temperature and radial position. For example, when the exposure temperature was 100 °C, the normalized concentration was 0.78, 0.81, 0.86, and 0.91 for 104 days’ immersion at *r* = 0.0 mm, 3.0 mm, 6.0 mm, and 7.75 mm. While the normalized concentration was close to half (0.49) for only 12 days’ immersion at *r* = 7.75 mm.

**Figure 13:**
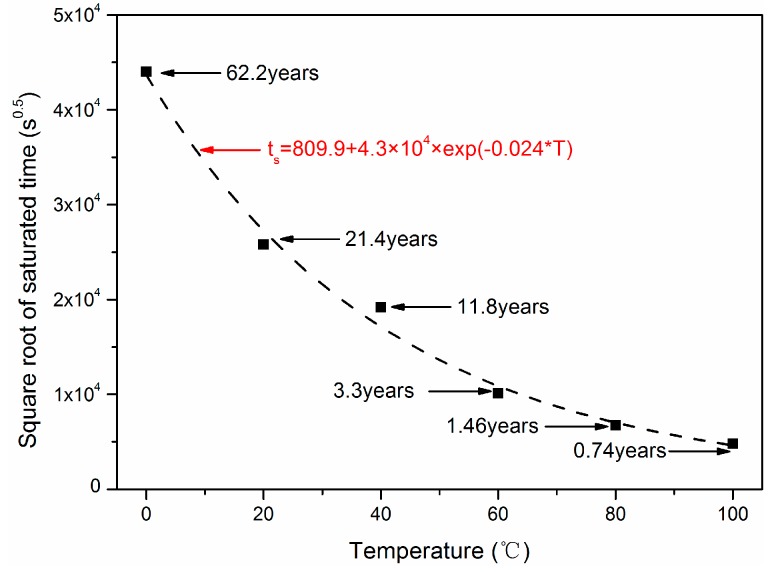
Dependence of saturation time on immersion temperature.

When the normalized concentration was one for *r* = 0.0 mm, the hybrid rod reached saturated condition and the saturated time *t*_s_ was obtained as shown in Figure 13. As shown, the saturated time presented on exponent distribution with immersion temperature *T*. When *T* = 20 °C and 100 °C, the saturated time *t*_s_ was 21.4 years and 0.74 years, respectively.

## 4. Conclusions

In the current work, the effect of high temperature and hydraulic pressure on the water absorption and distribution of the hybrid FRP rods was investigated experimentally and numerically. A speculative constant value (0.6) is assumed for the saturation water uptake for the unavailable weight gain at greater immersion times. With this value, fittings were performed for the four immersion cases through the Fickian law.

The following conclusions can be drawn based on the study:

(1) Water absorption curves of the hybrid FRP rod complied with Fickian law. The exposure temperatures and hydraulic pressure accelerated the water absorption and diffusion in the hybrid FRP rod. This was attributed to initial defects (such as resin matrix voids, fiber microcracks, and fiber/resin interface imperfections) inside the hybrid rod being rapidly filled by water molecules under the actions of exposure temperature and hydraulic pressure.
(2) By means of the diffusion mathematics theory, the equation of the water concentration distributions in the hybrid rod was deduced as a function of immersion time and temperature.
(3) The temperature and hydraulic pressure acceleration factor of water absorption were obtained, and the immersion time to reach saturation under various immersion conditions was predicted.

## Figures and Tables

**Table 1:** Average diffusivity coefficients of the hybrid FRP rods.

Temperatures (°C)	20	40	60	60/20 MPa
*D_av_* (m^2^/s)	1.26 × 10^−13^	2.30 × 10^−13^	8.14 × 10^−13^	11.17 × 10^−13^

**Table 2:** Diffusivity coefficients Dc of CFRP and D_G_ of GFRP.

Temperature (°C)	20	40	60	60/20 MPa
*D_C_* (m^2^/s)	1.39 × 10^−13^	2.55 × 10^−13^	9.01 × 10^−13^	12.36 × 10^−13^
*D_G_* (m^2^/s)	1.06 × 10^−13^	1.93 × 10^−13^	6.84 × 10^−13^	9.38 × 10^−13^

**Table 3:** Immersion time (*t*_start_, *t*_end_) of initial and saturated normalized concentration at *r* = 0, 3.0, 6.0, and 7.75 mm exposed at 20 °C, 40 °C, 60 °C, and 60 °C/20 MPa.

Conditions	20 °C		40 °C		60 °C		60 °C/20 MPa	
	*t*_start_ (day)	*t*_end_ (year)	*t*_start_ (day)	*t*_end_ (year)	*t*_start_ (day)	*t*_end_ (year)	*t*_start_ (day)	*t*_end_ (year)
*r* = 0 mm (CFRP)	362	21.4 *	198	11.8 *	56	3.3 *	41	2.4 *
*r* = 3.0 mm (CFRP)	231	21.2	126	11.6	35.5	3.3	26	2.4
*r* = 6.0 mm (CFRP/GFRP)	73	20.4	40	11.2	11	3.2	8	2.3
*r* = 7.75 mm (GFRP)	25	17.1	13	9.4	4	2.6	2	1.9

Note: the superscript “*” represented on the saturated time of HFRP rod.

**Table 4:** Maximum temperature and hydraulic pressure acceleration time for *r* = 0, 3.0 mm, 7.75 mm.

Conditions	*t* (C_40°C_/C_20°C_ = max) (day)	*t* (C_60°C_/C_20°C_ = max) (day)	*t* (C_60°C/20MPa_/C_60°C_ = max) (day)
*r* = 0.0 mm	127.3	111.7	20.8
*r* = 3.0 mm	71.8	62.5	11.6
*r* = 7.75 mm	12.2	11.0	2.3

## Appendix A

For a long cylinder in which diffusion was everywhere radial, the diffusion equation was shown as the following:

(A1) $$\frac{\partial C(r,t)}{\partial t}=\frac{1}{r}\frac{\partial }{\partial t}(rD\frac{\partial C(r,t)}{\partial r})$$

where *C*(*r,t*) was the concentration distribution, *r* was the radial position of the hybrid rod, *t* was the water absorption time, and *D* was the diffusivity coefficient; its value was *D_C_* for the CFRP core layer and *D_G_* for the GFRP shell layer.

By using the method of variables separation, the general solution of Equation (A1) was given in Equation (A2).

(A2) $$C(r,t)=u(r)\exp(-D\alpha^{2}t)$$

Here it should be noted that the hybridization of the GFRP shell layer and CFRP core does not affect the water absorption behavior of the hybrid rod, and the nonhybrid GFRP and CFRP rod follow a typical Fickian law. Substituting Equation (A2) into Equation (A1) yields Equation (A3):

(A3) $$\frac{d^{2}u(r)}{dr^{2}}+\frac{1}{r}\frac{du(r)}{dr}+\alpha^{2}u(r)=0$$

The solution form of Equation (A3) satisfied the zero-order Bessel function of the first kind. The derivation of the solution of Equation (A2) can be divided into three steps based on the initial (*C*(*r,t*), *t* = 0) and boundary conditions (*C*(*r,t*), *r* = *b*).

In the first step, the initial and boundary conditions were shown as the following:

(A4) $$\begin{array}{l} C=0,r=b,t\geq 0\\ C=f(r),a<r<b,t=0 \end{array}$$

where *f*(*r*) was the initial concentration distribution related to radial position *r* and the concentration of outermost shell (*r* = *b*) was zero. From the zero-order Bessel function of the first kind, the general solution of Equation (A2) was expressed as:

(A5) $$C(r,t)=\sum_{n=1}^{\infty }A_{n}J_{0}(\alpha_{n}r)\exp(-D\alpha_{n}^{2}t),\;a<r<b$$

where *A_n_* was the combination coefficient, *J*_0_ was the zero-order Bessel function of the first kind, and *α*_n_ was the *n*th root of the zero-order Bessel function. Substituting the initial condition of Equation (A4) into Equation (A5) yields:

(A6) $$f(r)=\sum_{n=1}^{\infty }A_{n}J_{0}(\alpha_{n}r),\;a<r<b$$

The *A_n_s* were determined by multiplying both sides of (A6) by $\int_{a}^{b}rJ_{0}(\alpha_{n}r)dr$ as follows:

(A7) $$A_{n}=\frac{2}{b^{2}J_{1}^{2}(b\alpha_{n})-a^{2}J_{1}^{2}(a\alpha_{n})}\int_{a}^{b}rf(r)J_{0}(r\alpha_{n})dr$$

where *J*_1_ was the first-order Bessel function of the first kind. It should be noted that the integration properties and integral continuity of the Bessel function were used during the deduction. Substituting Equation (A7) into Equation (A5), the concentration distribution *C*(*r,t*) was obtained as follows:

(A8) $$C(r,t)=2\sum_{n=1}^{\infty }\frac{J_{0}(\alpha_{n}r)}{b^{2}J_{1}^{2}(b\alpha_{n})-a^{2}J_{1}^{2}(a\alpha_{n})}\exp(-D\alpha_{n}^{2}t)\int_{a}^{b}rf(r)J_{0}(r\alpha_{n})dr,\;a<r<b$$

For the water absorption behavior of the hybrid rod, the initial concentration (*t* = 0) represented the dry condition and should be constant along the radial distribution. In the second step, the initial condition was modified as the following:

(A9) $$\begin{array}{l} C=0,r=b,t\geq 0\\ C=C_{1},a<r<b,t=0 \end{array}$$

Substituting Equation (A9) into Equation (A8) yields:

(A10) $$C(r,t)=2C_{1}\sum_{n=1}^{\infty }\frac{J_{0}(\alpha_{n}r)}{b^{2}J_{1}^{2}(b\alpha_{n})-a^{2}J_{1}^{2}(a\alpha_{n})}\exp(-D\alpha_{n}^{2}t)\int_{a}^{b}rJ_{0}(r\alpha_{n})dr,\;a<r<b$$

Here the integral of $\int_{a}^{b}rJ_{0}(\alpha_{n}r)dr$ was calculated as shown in Equation (A12) using the below equation:

(A11) $$\begin{array}{l} \frac{d}{dx}(x^{v}J_{v}(x))=x^{v}J_{v-1}(x)\\ xJ_{1}(x)=\int xJ_{0}(x)dx,\;\mathrm{when}\;v=1 \end{array}$$

(A12) $$\int_{a}^{b}rJ_{0}(r\alpha_{n})dr=\frac{1}{\alpha_{n}}(bJ_{1}(b\alpha_{n})-aJ_{1}(a\alpha_{n}))$$

Substituting Equation (A12) into Equation (A10), the concentration distribution was given as follows:

(A13) $$C(r,t)=2C_{1}\sum_{n=1}^{\infty }\frac{J_{0}(\alpha_{n}r)}{b^{2}J_{1}^{2}(b\alpha_{n})-a^{2}J_{1}^{2}(a\alpha_{n})}\frac{1}{\alpha_{n}}(bJ_{1}(b\alpha_{n})-aJ_{1}(a\alpha_{n}))\exp(-D\alpha_{n}^{2}t),\;a<r<b$$

For the hybrid rod, the boundary concentration (*r* = *b*) represents the water condition and should also be a constant. In the third step, the boundary condition was modified as the following:

(A14) $$\begin{array}{l} C=C_{0},r=b,t\geq 0\\ C=C_{1},a<r<b,t=0 \end{array}$$

When the initial and boundary conditions were determined as Equation (A14), the solution of Equation (A2) finally turns into

(A15) $$\begin{array}{l} C(r,t)=C_{0}(1-2\sum_{n=1}^{\infty }\frac{J_{0}(\alpha_{n}r)}{b^{2}J_{1}^{2}(b\alpha_{n})-a^{2}J_{1}^{2}(a\alpha_{n})}\frac{1}{\alpha_{n}}(bJ_{1}(b\alpha_{n})-aJ_{1}(a\alpha_{n}))\exp(-D\alpha_{n}^{2}t))+\\ 2C_{1}\sum_{n=1}^{\infty }\frac{J_{0}(\alpha_{n}r)}{b^{2}J_{1}^{2}(b\alpha_{n})-a^{2}J_{1}^{2}(a\alpha_{n})}\frac{1}{\alpha_{n}}(bJ_{1}(b\alpha_{n})-aJ_{1}(a\alpha_{n}))\exp(-D\alpha_{n}^{2}t),\;a<r<b \end{array}$$

Normalizing the Equation (A15) and letting $C(r,t)=\frac{C(r,t)-C_{0}}{C_{1}-C_{0}}$, it is reduced to:

(A16) $$\begin{array}{l} C(r,t)=1-2\sum_{n=1}^{\infty }\frac{J_{0}(\alpha_{n}r)(bJ_{1}(b\alpha_{n})-aJ_{1}(a\alpha_{n}))}{b^{2}J_{1}^{2}(b\alpha_{n})-a^{2}J_{1}^{2}(a\alpha_{n})}\frac{1}{\alpha_{n}}\exp(-D\alpha_{n}^{2}t)),\;a<r<b \end{array}$$

Equation (A16) shows the concentration distribution dependent on radial position, immersion time, and diffusivity coefficients. For the hybrid rod in this study, the radius (*a*) of core layer CFRP, thickness (*b* − *a*) of shell GFRP, and their diffusivity coefficients (*D_C_* and *D_G_*) were considered in Equation (A16). Finally, the concentration distribution of water uptake for the hybrid rod is obtained and expressed as the following:

(A17) $$C_{C}(r,t)=1-\frac{2}{a}\sum_{n=1}^{\infty }\frac{J_{0}(\alpha_{n}r)}{J_{1}^{}(a\alpha_{n})}\frac{1}{\alpha_{n}}\exp(-D_{C}\alpha_{n}^{2}t)),\;0<r<a$$

(A18) $$C_{G}(r,t)=1-2\sum_{n=1}^{\infty }\frac{J_{0}(\alpha_{n}r)(bJ_{1}(b\alpha_{n})-aJ_{1}(a\alpha_{n}))}{b^{2}J_{1}^{2}(b\alpha_{n})-a^{2}J_{1}^{2}(a\alpha_{n})}\frac{1}{\alpha_{n}}\exp(-D_{G}\alpha_{n}^{2}t)),\;a<r<b$$

where *C_C_*(*r,t*) and *C_G_*(*r,t*) were the concentration distributions of the CFRP core and GFRPs shell layer, respectively; *a*, *b* were the radii of core layer CFRP and the whole rod, respectively; *J*_0_, *J*_1_ were the zero and first-order Bessel function of the first kind, respectively; and *D_C_*, *D_G_* were the diffusivity coefficients of CFRP and GFRP, respectively.
